# Rest-activity rhythms and tract specific white matter lesions in older adults at risk for cognitive decline

**DOI:** 10.1038/s41380-022-01641-4

**Published:** 2022-06-28

**Authors:** Jake R. Palmer, Chenyu Wang, Dexiao Kong, Marcela Cespedes, Jonathon Pye, Ian B. Hickie, Michael Barnett, Sharon L. Naismith

**Affiliations:** 1 School of Psychology, The University of Sydney, Sydney, NSW Australia; 2 Brain and Mind Centre, The University of Sydney, Sydney, NSW Australia; 3 Charles Perkins Centre, The University of Sydney, Sydney, NSW Australia; 4 Department of Psychology, Macquarie University, Sydney, NSW Australia; 5 Sydney Neuroimaging Analysis Centre, Sydney, NSW Australia; 6 Australian e-Health Research Centre, CSIRO Health and Biosecurity, Herston, QLD Australia; 7 Susan Wakil School of Nursing and Midwifery, The University of Sydney, Sydney, NSW Australia; 8 NHMRC Centre of Research Excellence to Optimise Sleep in Brain Ageing and Neurodegeneration, Sydney, NSW Australia

**Keywords:** Neuroscience, Prognostic markers

## Abstract

White matter lesions (WMLs) are common in older adults and represent an important predictor of negative long-term outcomes. Rest-activity rhythm disturbance is also common, however, few studies have investigated associations between these factors. We employed a novel AI-based automatic WML segmentation tool and diffusion-weighted tractography to investigate associations between tract specific WML volumes and non-parametric actigraphy measures in older adults at risk for cognitive decline. The primary non-parametric measures of interest were inter-daily stability (IS), intra-daily variability and relative amplitude, with the anterior thalamic radiation (ATR), superior longitudinal fasciculus (SLF) and inferior longitudinal fasciculus (ILF) selected as tracts of interest. One hundred and eight participants at risk for cognitive decline (classified as experiencing subjective or objective cognitive decline) were included (mean age = 68.85 years, SD = 8.91). Of the primary non-parametric measures of interest, results showed that lower IS was associated with a greater likelihood of higher WML burden in the ATR (OR = 1.82, 95% CI [1.12,3.15]). Analysis of secondary non-parametric measures revealed later onset of the least active period to be associated with greater likelihood of high WML burden in the SLF (OR = 1.55, 95% CI [1.00,2.53]) and increased activity during the least active 5-h period to be associated with a greater likelihood of high whole-brain WML burden (OR = 1.83, 95% CI [1.06,3.47]). This study shows integrity of the ATR and SLF, and overall WML burden is linked to altered rest-activity rhythms in older adults at risk for cognitive decline, with those demonstrating altered rest-activity rhythms showing 50%-80% higher odds of having high WML burden.

## Introduction

Discrete white matter lesions (WMLs) have historically been the most widely investigated marker of white matter damage due to their ease of visualisation on commonly acquired magnetic resonance imaging (MRI) acquisitions. Cross-sectionally, WMLs in older adults have been associated with vascular risk factors [1, 2], late-life depression [3], and risk of stroke and cognitive impairment [4]. Longitudinally, cerebrovascular disease and WMLs have been shown to contribute to negative long-term outcomes including functional disability [5], cerebral amyloid angiopathy [6], and increased risk of dementia and mortality [4, 7].

One under-explored factor that may be related to WMLs and is common in older adults [8] is alterations in 24-h rest-activity rhythms. Disruption to rest-activity rhythms that are common in older adults at risk for dementia, including reduced rest-activity amplitude and increased fragmentation of the rest-activity rhythm [9], which have been associated with depression [10], cognitive impairment [11], markers of Alzheimer’s disease (AD) pathology [12] and hippocampal atrophy [13]. Non-parametric approaches have been applied to describe features of the 24-h rhythm, including fragmentation measured by intra-daily variability (IV), synchronisation to the 24-h light–dark cycle measured by inter-daily stability (IS) and relative amplitude (RA; amplitude of activity during active period relative to rest) [14]. In contrast to classic methods for actigraphy analysis such as cosinor analysis, which assume data is normally distributed and fits a sinusoidal curve (therefore referred to as parametric approaches), non-parametric measures make no assumptions regarding the distribution of the rest-activity data (see Neikrug et al. [15] for discussion).

Taken together, there is converging evidence linking WMLs and rest-activity rhythm disturbance to poorer long-term outcomes. However, to date, it is unclear whether WML burden and rest-activity disturbance are related in at risk older adults, with only a handful of studies having investigated potential associations. In addition, previous studies have primarily examined whole-brain WMLs, potentially missing differential regional contributions of WML burden. For example, Zuurbier et al. [16] found fragmented 24-h rhythms to be associated with WML burden, independent of total sleep time and quality. Oosterman et al. [17] additionally investigated regional deep and periventricular WML volumes and found deep frontal WML volumes to be associated with the amplitude of the rest-activity rhythm and synchronisation to the 24-h light–dark cycle. The identification of more specific regional contributions of WMLs is likely to provide deeper insights into the neurocircuitry underpinning these relationships in comparison to examining whole WML volumes. For example, Palhaugen et al. [2] recently demonstrated that frontal WMLs were associated with vascular risk while more posterior WML burden was related to greater likelihood of being *β*-amyloid positive. Based on findings such as these, it is plausible that tract specific white matter disruption may also be associated with rest-activity functioning in older adults who are at risk for dementia, however, this remains unexplored.

Currently, manual segmentation is the gold standard for quantification of WML burden, however, this approach is time consuming, labour intensive and susceptible to subjective rater effects, making it difficult to scale for large research studies or routine clinical use [18]. Recently, there has been interest in fully automatic WML segmentation methods [18, 19]. One such method is a recently proposed framework developed for the segmentation of WMLs in multiple sclerosis (MS) patients, which is based on a generative adversarial network (GAN) and has been shown to outperform several other fast, automatic AI-based WML segmentation frameworks [20]. Application of this method for the quantification of WMLs in older adults raises the potential for fast, consistent, and accurate objective measurement of WML burden in large clinical studies, research trials and clinical practice.

We implemented MS-GAN for automatic quantification of WML volumes to investigate associations between non-parametric actigraphy measures of rest-activity rhythms and WML volumes in a sample of older adults at risk for cognitive decline [21–23].

1. The primary aim was to determine whether tract specific WML volumes are related to three key measures of non-parametric rest-activity rhythms: IS, IV and RA.
2. A secondary aim was to investigate whether associations differed between white matter bundles. The anterior thalamic radiation (ATR) was selected as the primary tract of interest given previous literature implicating frontal WML volumes in rest-activity disruption, as well as its potential role in propagation of sleep signals from the thalamus [24, 25]. The superior longitudinal fasciculus (SLF) was also included given its role as a major association pathway. The inferior longitudinal fasciculus (ILF) was also selected as rest-activity fragmentation has been implicated in hippocampal atrophy [14] and the ILF has been shown to be a major source of connectivity to the hippocampus [26]. Based on previous literature [17], we predicted that WML volume in the ATR would be related to IS and RA.
3. An exploratory aim was to investigate additional non-parametric measures of actigraphy, including mean activity during the least active 5-h period (L5) and most active 10-h period (M10), as well as the start time of the least active period, as associations between these measures and WMLs have not been previously investigated.
4. Finally, we also aimed to validate the MS-GAN automatic segmentation method that was trained in patients with MS, against gold-standard manual WML segmentation for use in older adult populations.

## Methods

### Participants

All participants (aged 50 years or older) were recruited from the Healthy Brain Ageing Clinic at the Brain and Mind Centre, The University of Sydney. The sample included in this study represents a subset of participants recruited through the Healthy Brain Ageing Clinic to a larger, ongoing cohort study who had undergone clinical, actigraphic and MRI assessment. Exclusion criteria for the clinic are having a primary language other than English, Mini-Mental State Examination (MMSE) [27] less than 24, a diagnosis of dementia, significant neurological disorder (e.g., Parkinson’s disease or epilepsy), head injury or psychiatric illness, or a history of substance abuse. For this sub-study, we additionally required participants to have completed at least 7 days of actigraphy recording and MRI. All participants provided written informed consent before participation in the study and all research activities were approved by The University of Sydney Human Research Ethics Committee.

### Clinical assessment

As described in detail elsewhere [28], all participants underwent detailed medical, psychological, and neuropsychological assessment. Although a full neuropsychological assessment was conducted, we report the MMSE score as a measure of global cognition for descriptive purposes. A semi-structured medical assessment was conducted by a geriatrician and included detailed medical history, medical burden (Cumulative Illness Rating Scale-Geriatric version [29]) and measurement of participants’ height and weight to calculate body mass index (BMI). A vascular risk index was derived to indicate presence of the following known vascular risk factors (yes/no): high blood pressure, hypercholesterolaemia, history or current smoking, heart disease and diagnosis of diabetes (max score = 5). All participants also completed the Pittsburgh Sleep Quality Index (PSQI) [30] and the Geriatric Depression Scale-15 item (GDS-15) [31].

Classifications of mild cognitive impairment (MCI) were given by consensus rating (medical specialist and two neuropsychologists) according to established criteria that required objective evidence of cognitive impairment with intact functional abilities [32]. Participants who did not meet criteria for MCI were classified as experiencing subjective cognitive decline (SCD) as all participants sought referral to the clinic for concerns regarding their cognition.

### Actigraphy

All participants were asked to undergo 14 days of actigraphy recording within three months of undergoing MRI. Participants wore an actigraphy watch on their non-dominant wrist (Actiwatch Spectrum, Minimitter-Respironics, OR) and completed a daily sleep diary [33]. The ‘Multivariate Imputation by Chained Equations’ (*mice*) R package (version 3.9.0) [34] was used to characterise and impute missing actigraphy data (median missing data 2.16%, maximum 9.42%). Further details are included in Supplementary Material.

### Non-parametric actigraphy analysis

Non-parametric actigraphy measures were derived with the *nparACT* R package (version 0.8) [35], including IV, IS, RA, M10, L5 and L5 start time [36]. These measures are reviewed elsewhere [15]. Briefly, IV quantifies the rate of shifting between rest and activity in adjacent hourly bins, with a higher statistic indicating higher variability indicative of rhythm fragmentation. IS determines the similarity of daily activity patterns to the average daily activity pattern and therefore represents synchronisation to the 24-h light–dark cycle, with higher values representing greater synchronisation. RA is derived from activity measurement during the L5 and M10 as the difference between L5 and M10 divided by their sum, with greater activity during active periods and lower activity during rest being indicative of a healthy 24-h rhythm. Finally, L5 start time represents the start time of the least active period.

### MRI acquisition

All MRI was acquired on a 3T GE Discovery MR750 scanner (GE Medical Systems, Milwaukee, WI, USA). Both axial 2D (4.0 mm slices) and sagittal 3D (1.2 mm slices) FLAIR acquisitions were included, along with 3D T1-weighted images (0.9 mm isotropic). A subset of participants also completed diffusion-weighted imaging (DWI; 64 slices, 2.5 mm thickness, 32 gradient directions, *b* = 1000 s/mm^2^, with two *b* = 0 s/mm^2^ volumes). All images were acquired with an 8-channel phased array head coil (see Supplementary Material for detailed acquisition parameters).

### Automatic WML segmentation

We employed MS-GAN previously trained in individuals with MS [20] to automatically quantify WML volumes for each participant. Lesion masks were generated in a fully automated manner from both T1-weighted and FLAIR images, which were brain extracted [37] and co-registered (FLIRT; FSL, version 6.0.0, [38]). All brain segmentations were visually inspected.

### MS-GAN validation

The development of MS-GAN is detailed elsewhere [20] and has been shown to outperform other popular deep-learning architectures. However, given this is the first application of the method in an older adult population, we validated the segmentations produced by MS-GAN against manual segmentations. A random sample of 50 FLAIR scans that were not included in the current study but who had previously attended the Healthy Brain Ageing Clinic were selected (25 with 2D FLAIR and 25 3D FLAIR as described above), regardless of clinical. All participants’ WMLs were manually segmented by agreement by two trained researchers using ITKSNAP (www.itksnap.org). The performance of MS-GAN was compared to manual segmentation using voxel-wise Dice coefficients [39, 40] and Bland-Altman plots [41]. Normality of differences between methods [42] were assessed via the Shapiro–Wilk test.

### Tract-level WML volumes

**Fig. 1:**
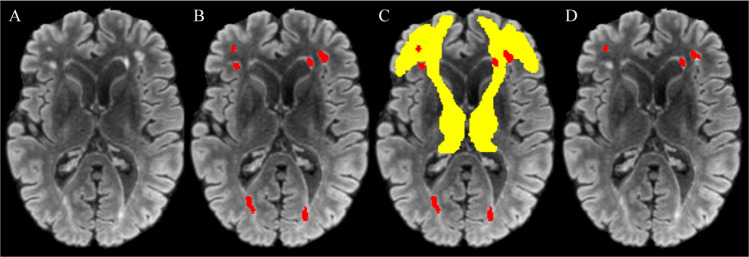
Steps to derive tract specific WML volumes. MS-GAN was applied to **A** each participants FLAIR scan to derive **B** binary whole brain lesion masks (red). **C** The intersection of tract masks computed from the DWI template (non-linearly registered to subject space; ATR shown in yellow) and the whole brain lesion masks was calculated to derive **D** the final tract specific binary lesion masks for statistical analysis.

A study-specific fibre orientation distribution (FOD) template was generated from a random subset of 30 participants who also had DWI. All DWI data were processed with MRtrix (version 3.0_RC3) [43]. Pre-processing included de-noising [44]; eddy current correction with FSL’s *eddy* [45]; N4 bias field correction ([46]) and global intensity normalisation. Two-tissue constrained spherical deconvolution (CSD) was used to model white matter FODs [47] from which the template was generated. Tract masks were generated from the FOD template with TractSeg (version 2.1.1) [48] and non-linearly registered from template space to each FLAIR image in subject space [49] to derive tract-level WML volumes (Fig. 1). Lesion volumes from each hemisphere were averaged.

### Statistical analysis

Bilateral WML volumes were severely skewed and therefore split at the median into ‘high’ or ‘low’ volumes (Table 1). Bayesian logistic regression in R (version 4.0.0 [50, 51]) was used to investigate relationships between non-parametric actigraphy measures and level of WML volume in each bilateral tract. To address differences in scale of measurement, continuous independent variables were converted to *z*-scores. Age, sex, and FLAIR scan type were included as covariates in all models. Given ‘high’ and ‘low’ whole-brain WML groups differed in BMI (Table 2), any model that identified an effect of a non-parametric actigraphy measure on WML volume was repeated with BMI included as an additional covariate. Code available at https://github.com/jakepalmer.

**Table 1:** Summary statistics.

	Missing	Overall
Clinical characteristics		
*N*		108
Age (years)	0	68.85 (8.91)
Sex, female (%)	0	63 (58.33)
Scan type, 2D (%)	0	40 (37.04)
MMSE	0	29.00 [28.00,30.00]
GDS-15	5	2.00 [1.00,4.00]
PSQI total score	6	7.13 (3.77)
BMI	5	26.10 [23.04,30.60]
Antidepressants (%)^a^	0	24 (22.43)
CIRS-G	8	4.00 [2.00,6.00]
Vascular risk index, (%)	7	
0		37 (36.63)
1		38 (37.62)
2		20 (19.80)
3		6 (5.94)
Actigraphy derived measures		
Total sleep time (min)	0	436.62 (64.26)
WASO (min)	0	50.19 (38.62)
Inter-daily stability	0	0.53 (0.13)
Intra-daily variability	0	0.80 (0.22)
Relative amplitude	0	0.90 [0.85,0.93]
L5	0	6.34 [4.32,10.30]
M10	0	129.77 (47.58)
L5 start time (24-h)	0	00:45 (01:21)
WML volumes (mm^3^*)*		
Anterior thalamic radiation	0	376.80 [74.68,972.06]
Superior longitudinal fasciculus	0	167.63 [21.61,587.84]
Inferior longitudinal fasciculus	0	180.16 [89.67,315.80]
Whole brain	0	3609.55 [1565.72,7817.48]

Mean (SD); Median [IQR].

*MMSE* Mini-Mental State Examination, *GDS-15* Geriatric Depression Scale-15 item, *PSQI* Pittsburgh Sleep Quality Index, *BMI* body mass index, *CIRS-G* Cumulative Illness Rating Scale-Geriatric version, *WASO* wake after sleep onset, *L5*=mean activity during least active 5-h period, *M10* mean activity during most active 10-h period, *WML* white matter lesions.

^a^SSRI = 10, SNRI = 10, TCA = 2, not disclosed = 2.

**Table 2:** Summary statistics by high and low whole-brain WML volume groups.

	High	Low	*p* value
Clinical characteristics			
* n*	54	54	-
Age (years)^a^	73.52 (7.63)	64.19 (7.60)	<0.001
Sex, female (%)^b^	32 (59.26)	31 (57.41)	1.000
Scan type, 2D (%)^b^	25 (46.30)	15 (27.78)	0.073
MMSE^c^	29.00 [29.00,30.00]	29.00 [28.00,30.00]	0.300
GDS-15^c^	2.00 [0.00,4.00]	2.00 [1.00,4.00]	0.526
PSQI total score^a^	6.39 (3.68)	6.86 (3.86)	0.530
BMI^c^	27.30 [24.96,30.60]	25.00 [21.99,29.43]	0.030
Antidepressants (%)^b^	13 (24.07)	11 (20.75)	0.857
CIRS-G^c^	5.00 [3.00,6.00]	3.00 [2.00,6.00]	0.063
Vascular risk index, (%)^d^			0.813
0	18 (37.50)	19 (35.85)	
1	16 (33.33)	22 (41.51)	
2	11 (22.92)	9 (16.98)	
3	3 (6.25)	3 (5.66)	
Actigraphy derived measures			
Total sleep time (min)	424.67 (69.61)	448.57 (56.59)	0.055
WASO (min)	54.34 (43.98)	46.04 (32.27)	0.266
Inter-daily stability	0.51 (0.13)	0.55 (0.12)	0.093
Intra-daily variability	0.83 (0.22)	0.78 (0.22)	0.215
Relative amplitude	0.89 [0.83,0.93]	0.91 [0.86,0.94]	0.078
L5	7.21 [4.32,11.91]	6.12 [4.33,9.02]	0.291
M10	124.02 (47.77)	135.53 (47.14)	0.210
L5 start time (24-h)	1477.00 (86.41)	1457.07 (53.65)	0.153

Mean (SD) unless otherwise indicated; Median [IQR].

*MMSE* Mini-Mental State Examination, *GDS-15* Geriatric Depression Scale-15 item, *BMI* Body Mass Index, *CIRS-G* Cumulative Illness Rating Scale-Geriatric version, *WASO* Wake After Sleep Onset, *L5* Mean activity during least active 5-h period, *M10* Mean activity during most active 10-h period, *WML* White matter lesions.

^a^*T*-test.

^b^Chi-square test.

^c^Kruskal–Wallis test.

^d^Fisher’s exact test.

Informative Gaussian priors were selected based on previous evidence where available, otherwise conservative weakly informative Gaussian priors were used (see Supplementary Material). Marginal posterior distributions were computed with *rstanarm* (version 2.19.3 [52]). These posterior distributions represent the range of possible values of the regression coefficients supported by the data and are described by a point estimate (the median) and 95% credible intervals (CI), which we also report as odds ratios (OR). The strength of evidence can be evaluated by determining whether a parameter value of zero falls within the 95% CI, with intervals that do not include zero being indicative of evidence supporting an effect.

## Results

### MS-GAN validation

**Fig. 2:**
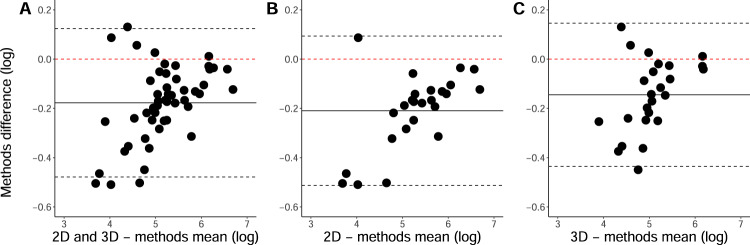
Bland–Altman plots comparing manual and MS-GAN segmentations. Bland–Altman plots comparing manual WML segmentation with MS-GAN automatic segmentations for **A** 2D and 3D FLAIR scans together, **B** 2D FLAIR scans only and **C** 3D FLAIR scans only. Values are log-transformed lesion volumes (mm^3^). The solid black line shows the mean of differences between methods, with dashed black lines showing the limits of agreement (±1.96 standard deviations).

No manual edits were made to the automatically generated masks. The voxel-wise Dice coefficient for all validation FLAIR scans (2D and 3D combined) was 0.73 and, when considered separately, both 2D and 3D scans had Dice coefficients of 0.71. The mean subject-wise Dice coefficient for all scans were 0.54, 0.52 for 2D scans and 0.56 for 3D scans. Lower subject-wise Dice coefficients are expected as a small number of participants with larger discrepancies can bias the estimate downwards. Given non-normality of the differences between automatic and manual segmentation (*W* = 53.13, *p* < 0.001), log-transformed WML volumes were used for Bland-Altman plots (Fig. 2).

The voxel-wise Dice coefficients demonstrate an acceptable level of agreement between manual and MS-GAN segmentations, with Fig. 1 showing that MS-GAN results in marginally higher segmentation volumes relative to manual segmentation. This effect is slightly more apparent for 2D scans, potentially due to greater imaging artefacts, however, visual inspection of MS-GAN lesion masks would be sufficient to identify any problematic cases.

### Participant characteristics

One hundred and nine participants with at least 7 days of actigraphy recording (median = 14, range = 7–14) and MRI were recruited. One participant was excluded due to an incidental finding on MRI, meaning a total of 108 were included for analysis (mean age = 68.85 years, SD = 8.91; Table 1). Overall, participants did not report elevated depressive symptoms, reported mild sleep disturbance, and had low levels of medical burden with only 22% currently taking antidepressants. Seventy-three per cent of the sample met clinical criteria for MCI, with the remaining participants classified as SCD due to the sample seeking assessment for concerns regarding their cognition. As the participants with MCI did not differ from those with SCD on any WML volumes, actigraphy measures or any clinical measure aside from MMSE (Table S1), all participants were subsequently considered as a single group.

### Tract-level WML volumes

Tract-level WML volumes for the ATR, SLF and ILF were included as the dependent variables in separate logistic regression models, with posterior estimates for all following models presented in Table 3.

**Table 3:** Logistic regression marginal posterior distributions summary.

	Median (95% CI)	with BMI
Anterior thalamic radiation		
Inter-daily stability	−0.60 (−1.15,−0.11)	−0.56 (−1.10, −0.07)
Relative amplitude	−0.05 (−0.52,0.41)	–
Intra-daily variability	0.24 (−0.21,0.70)	–
L5	0.09 (−0.36,0.57)	–
M10	−0.31 (−0.80,0.15)	–
L5 start time	0.43 (−0.02,0.92)	0.38 (−0.10,0.89)
Superior longitudinal fasciculus		
Inter-daily stability	−0.40 (−0.91,0.06)	−0.32 (−0.84,0.16)
Relative amplitude	−0.12 (−0.61,0.32)	–
Intra-daily variability	−0.00 (−0.44,0.43)	–
L5	0.25 (−0.19,0.77)	–
M10	−0.07 (−0.52,0.39)	–
L5 start time	0.44 (0.00,0.93)	0.38 (−0.10,0.87)
Inferior longitudinal fasciculus		
Inter-daily stability	−0.09 (−0.50,0.31)	–
Relative amplitude	0.11 (−0.30,0.53)	–
Intra-daily variability	−0.05 (−0.44,0.35)	–
L5	−0.16 (−0.58,0.24)	–
M10	−0.22 (−0.65,0.19)	–
L5 start time	0.09 (−0.30,0.48)	–
Whole brain		
Inter-daily stability	−0.61 (−1.19,−0.10)	−0.58 (−1.15,−0.07)
Relative amplitude	−0.28 (−0.84,0.19)	–
Intra-daily variability	0.23 (−0.22,0.71)	–
L5	0.60 (0.06,1.25)	0.75 (0.13,1.49)
M10	−0.04 (−0.51,0.44)	–
L5 start time	0.34 (−0.12,0.83)	–

Median = Median of marginal posterior distribution; All models include age, sex, and FLAIR scan type (2D or 3D) as covariates.

*CI* Credible Interval.

### Primary non-parametric actigraphy measures of interest

For the primary non-parametric actigraphy measures of interest, strong evidence was found for poorer synchronisation to the 24-h light–dark cycle (lower IS) being associated with a greater likelihood of having a higher WML volume in the ATR (OR = 1.82, 95% CI [1.12,3.15]). This effect remained when BMI was included in the model (OR = 1.75, 95% CI [1.07,3.02]). Weak evidence was also found for the same effect in the SLF (OR = 1.49, 95% CI [0.94,2.47]), however, there was no evidence supporting this effect when BMI was included (OR = 1.38, 95% CI [0.85,2.31]). There was no evidence supporting an effect of IS in the ILF or an effect of RA in any of the ATR, ILF or SLF.

### Secondary non-parametric actigraphy measures

Of the secondary non-parametric actigraphy measures, only L5 start time was found to be related to WML volumes. Specifically, later start time of the L5 period was associated with a greater likelihood of having higher WML volume in both the ATR and the SLF (OR = 1.54, 95% CI [0.98,2.51] and OR = 1.55, 95% CI [1.00,2.53] respectively), however these effects did not remain when BMI was included (OR = 1.46, 95% CI [0.91,2.43] and OR = 1.46, 95% CI [0.91,2.40], respectively). There was no evidence supporting an effect of IV, L5 or M10 in the ATR, ILF or SLF.

### Whole-brain WML volume

#### Primary non-parametric actigraphy measures of interest

Logistic regressions revealed that poorer synchronisation to the 24-h light–dark cycle (lower IS) was associated with a greater likelihood of having a higher level of whole-brain WML volume (OR = 1.84, 95% CI [1.11,3.30]), with the effect remaining when BMI was also included (OR = 1.79, 95% CI [1.07,3.15]). There was again no evidence supporting an effect of RA.

#### Secondary non-parametric actigraphy measures

Of the secondary non-parametric measures, greater average activity during the L5 period was associated with a higher likelihood of higher whole brain WML volume (OR = 1.83, 95% CI [1.06,3.47]), with evidence supporting this effect strengthened by the inclusion of BMI in the model (OR = 2.12, 95% CI [1.14,4.42]). There was no evidence supporting an effect of IV, M10 or start time of the L5 period.

## Discussion

This study utilised a novel method for measuring WMLs within key tracts to examine for the first time how tract specific WML volumes relate to rest-activity rhythms in older adults at risk for cognitive decline and dementia. We found a strong association between IS and ATR WML volume, such that poorer synchronisation to the 24-h light–dark cycle was associated with greater likelihood of higher WML volume. This study also identified novel relationships between later onset of the least active period and WML burden in the ATR and SLF, along with greater average activity during the least active period and higher whole brain WML burden. Contrary to previous studies (e.g., [17]), no evidence was found for an association between RA and WMLs. Finally, we demonstrated the validity of a novel AI-based automatic WML segmentation method originally trained on participants with MS.

The novel association between lower IS and WML burden within the ATR revealed in this study advances understanding of the relationship between white matter pathology and rest-activity rhythms in older adults. Given IS was also associated with overall WML burden, it is also aligned with prior work demonstrating associations between poorer synchronisation to the 24-h light–dark cycle and WML burden [17].

Although there is converging evidence highlighting associations between disrupted rest-activity rhythms and WML burden, the mechanism by which rest-activity rhythms contribute to the development WMLs, or vice versa, remains unclear. Frontal WMLs may disrupt inputs to the suprachiasmatic nucleus (SCN) [17], which regulates circadian rhythms throughout the central and peripheral systems [53]. Alternatively, WMLs may disrupt brain networks associated with SCN output. For example, the SCN has extensive efferent connections with the hypothalamus, which subsequently projects to key nodes of the ascending arousal system and the thalamus [54], which is critical in maintaining the balance of sleep and circadian regulation [24]. WMLs affecting thalamic projections, such as the ATR, may disrupt this balance and contribute to alterations in rest-activity rhythms, contributing to a cycle of unstable rest-activity rhythms and poor overnight sleep quality. Importantly, cross-sectional studies such as the current one cannot identify causal relationships, with longitudinal studies required to delineate the direction of effects observed here.

Interestingly, our data also showed more nocturnal arousals during the least active period of sleep (i.e., higher L5) were associated with an approximately 80% increased odds of greater whole-brain WMLs. This is somewhat aligned with the study by Thurston et al. [55] who found greater nocturnal arousals measured by ‘wake after sleep onset’, were associated with increased WML volumes. The association revealed between L5 and whole-brain WML volume but not WML volume in any of the tracts of interest potentially indicates a differential effect of frontal WMLs relative to more posterior WMLs not captured in the tracts of interest included here. Palhaugen et al. [2] recently suggested frontal WMLs to be related to vascular risk, while more posterior WMLs were related to AD pathology. This is also consistent with Davatzikos et al. [56] who showed cognitively unimpaired individuals with greater AD-like atrophy had greater posterior periventricular white matter abnormalities than those with low AD-like atrophy. In this context, the current findings suggest frontal WML volumes may be related to rest-activity rhythm disruption, while more posterior WMLs captured in the whole brain WML volumes (that may be preferentially associated with AD pathology) may relate to greater nocturnal awakenings. Anatomical specificity of WML burden warrants further investigation as it may represent an early marker of pathological trajectories.

The current study should be interpreted in the context of some limitations. First, the tract masks used to identify tract specific WMLs were produced from a study-specific DWI template. Although all registrations were inspected, this method does not capture subtle inter-subject variability of white matter architecture. Second, the binary tract masks do not fully account for WMLs affecting multiple fibre bundles in regions of crossing fibres. Third, the current study did not incorporate a measure of sleep-disordered breathing. Although significant regression models were repeated additionally controlling for BMI, which is a strong predictor of sleep-disordered breathing [57], the fact BMI was found to differ between high and low whole-brain WML groups suggests it may have contributed to the relationships observed here. Finally, AD biomarkers were not available for this sample. Future studies should investigate the contribution of neuropathology to regional WML burden and rest-activity rhythms.

## Conclusion

This study provides novel evidence for associations between anatomical location of WMLs and features of rest-activity rhythms in older adults. Longitudinal studies examining the direction of these relationships are now required as both poor white matter health and rest-activity rhythm disturbance represent potentially modifiable risk factors for cognitive decline. Identifying which of these factors drive the relationship will provide important insight for directing targeted interventions aimed at altering the course of decline in older adults.

## Supplementary information

Supplementary material
