# Supplementary material for: Rest-activity rhythms and tract specific white matter lesions in older adults at risk for cognitive decline
Source: Mol Psychiatry. 2022 Jun 28;27(8):3410–6. doi: 10.1038/s41380-022-01641-4 (PMC9708592; doi:10.1038/s41380-022-01641-4)
Supplement: Supplementary file 1 — Supplementary material [file 41380_2022_1641_MOESM1_ESM.docx]

Rest-activity rhythms and tract specific white matter lesions in older adults at risk for cognitive decline

Jake R. Palmer *^a,b,c,d^*, Chenyu Wang *^b,e^*, Dexiao Kong *^a,b,c^*, Marcela Cespedes *^f^* , Jonathon Pye *^a,g^*,

Ian B. Hickie *^b^*, Michael Barnett *^b,e^*, Sharon L. Naismith *^a,b,c,h*^*

*^a^* School of Psychology, The University of Sydney, Sydney NSW, Australia

*^b^* Brain and Mind Centre, The University of Sydney, Sydney NSW, Australia

*^c^* Charles Perkins Centre, The University of Sydney, Sydney NSW, Australia

*^d^* Department of Psychology, Macquarie University, Sydney NSW, Australia

*^e^* Sydney Neuroimaging Analysis Centre, Sydney NSW, Australia

*^f^* Australian e-Health Research Centre, CSIRO Health and Biosecurity, Herston QLD, Australia

*^g^* Susan Wakil School of Nursing and Midwifery, The University of Sydney, Sydney NSW,

Australia

*^h^* NHMRC Centre of Research Excellence to Optimise Sleep in Brain Ageing and

Neurodegeneration

*Corresponding author:

Level 2, Building G, Brain and Mind Centre, 100 Mallet Street, Camperdown, NSW 2050 Australia

E-mail: [sharon.naismith@sydney.edu.au](mailto:sharon.naismith@sydney.edu.au)

# **Actigraphy**

Sleep diary was integrated with actigraphy-measured activity and light levels to define sleep onset and offset times, with the Actiware 5.0 software then used to determine total sleep time and wake after sleep onset (WASO). The wake threshold was set at a medium sensitivity of 40 counts per epoch, with 30 second epochs. Data were missing due to bathing and other activities involving the potential exposure of the device to water, as participants were instructed to remove the device. Before imputation, the epoch-by-epoch data was trimmed to include only complete 24-hour periods of recording, meaning the first and last day of the recording period were excluded. The ‘Multivariate Imputation by Chained Equations’ (*mice*) R package (version 3.9.0) [1] was used to characterise and impute missing actigraphy data using the random forest method including white light (in lux) and accelerometer counts (30-second epochs) and 10 imputations.

# **Bayesian logistic regression priors**

Based on previous literature [2–5], informative Gaussian priors were selected for IS, RA and M10 (mean = -0.5, SD = 1), as well as for IV and L5 (mean = 0.5, SD = 1). As no previous literature has examined associations between L5 start and WMLs, a conservative informative Gaussian prior centered on zero was selected (mean = 0, SD = 1). Informative Gaussian priors were also chosen for age (mean = 1, SD = 1) and BMI (mean = 0.5, SD = 1) given the well-established associations between increasing age [6, 7], vascular risk factors such as BMI [8], and WML burden. Finally, weakly-informative (relative to the scaled range of parameter estimates expected) Gaussian priors (mean = 0, SD = 2.5) were selected for sex and for scan type as this was the first application of MS-GAN to both 2D and 3D FLAIR scans.

# **References**

1. Buuren S van, Groothuis-Oudshoorn K. mice: Multivariate imputation by chained equations in R. *J Stat Softw*. 2011;45:1–67.
2. Oosterman J, Harten B van, Vogels R, Gouw A, Weinstein H, Scheltens P, et al. Distortions in rest-activity rhythm in aging relate to white matter hyperintensities. *Neurobiol Aging*. 2008;29:1265–1271.
3. Zuurbier LA, Ikram MA, Luik AI, Hofman A, Van Someren EJW, Vernooij MW, et al. Cerebral small vessel disease is related to disturbed 24-h activity rhythms: A population-based study. *Eur J Neurol*. 2015;22:1482–1487.
4. Torres ER, Strack EF, Fernandez CE, Tumey TA, Hitchcock ME. Physical activity and white matter hyperintensities: A systematic review of quantitative studies. *Prev Med Reports*. 2015;2:319–325.
5. Thurston RC, Wu M, Aizenstein HJ, Chang Y, Barinas Mitchell E, Derby CA, et al. Sleep characteristics and white matter hyperintensities among midlife women. *Sleep*. 2020;43:1–7.
6. Habes M, Pomponio R, Shou H, Doshi J, Mamourian E, Erus G, et al. The Brain Chart of Aging: Machine-learning analytics reveals links between brain aging, white matter disease, amyloid burden, and cognition in the iSTAGING consortium of 10,216 harmonized MR scans. *Alzheimer’s Dement*. 2021;17:89–102.
7. Prins ND, Scheltens P. White matter hyperintensities, cognitive impairment and dementia: an update. *Nat Rev Neurol*. 2015;11:157–165.
8. Debette S, Seshadri S, Beiser A, Au R, Himali JJ, Palumbo C, et al. Midlife vascular risk factor exposure accelerates structural brain aging and cognitive decline. *Neurology*. 2011;77:461–468.

Table 1: Summary statistics by cognitive classification

|  | MCI | SCD |
| --- | --- | --- |
| *Clinical Characteristics* |  |  |
| n^**^ | 79 | 29 |
| Age (years) | 69.42 (8.97) | 67.31 (8.73) |
| Sex, female (%) | 47 (59.49) | 16 (55.17) |
| Scan type, 2D (%) | 33 (41.77) | 7 (24.14) |
| MMSE^*^ | 29.00 [28.00,30.00] | 30.00 [29.00,30.00] |
| GDS-15 | 2.00 [1.00,4.00] | 2.00 [1.00,4.25] |
| PSQI total score | 6.84 (3.75) | 6.07 (3.79) |
| BMI | 25.56 [22.94,29.12] | 26.50 [24.80,31.40] |
| Antidepressants (%)^1^ | 58 (74.36) | 25 (86.21) |
| CIRS-G | 4.00 [2.00,6.00] | 4.00 [3.00,5.25] |
| Vascular risk index, (%) |  |  |
| 0 | 27 (36.49) | 10 (37.04) |
| 1 | 26 (35.14) | 12 (44.44) |
| 2 | 15 (20.27) | 5 (18.52) |
| 3 | 6 (8.11) | 0 (0.00) |
| *Actigraphy Derived Measures* |  |  |
| Total Sleep Time (mins) | 436.16 (58.55) | 437.83 (78.60) |
| WASO (mins) | 50.24 (36.24) | 50.06 (45.18) |
| Inter-daily Stability | 0.53 (0.12) | 0.52 (0.14) |
| Intra-daily Variability | 0.82 (0.22) | 0.76 (0.21) |
| Relative Amplitude | 0.90 [0.84,0.93] | 0.91 [0.86,0.95] |
| L5 | 7.01 [4.40,10.82] | 5.29 [3.82,9.09] |
| M10 | 131.23 (51.80) | 125.81 (34.00) |
| L5 start time (24-hour) | 00:43 (00:12) | 00:30 (00:16) |
| *WML volumes (mm*^3^*)* |  |  |
| Anterior Thalamic Radiation | 408.01 [116.56,975.07] | 277.50 [45.04,776.88] |
| Superior Longitudinal Fasciculus | 170.93 [21.37,607.05] | 164.34 [22.52,435.99] |
| Inferior Longitudinal Fasciculus | 173.24 [89.27,311.53] | 204.12 [113.68,352.31] |
| Whole Brain | 3757.04 [1589.74,7841.19] | 3199.94 [1569.70,7631.64] |

Note: Mean (SD); Median [IQR];^∗^*p<*.05; ^∗∗^*p<*.001; ^1^SSRI = 10, SNRI = 10, TCA = 2, not disclosed = 2; MMSE = Mini-Mental State Examination; GDS-15 = Geriatric Depression Scale–15 item; PSQI = Pittsburgh Sleep Quality Index; BMI = Body Mass Index; CIRS-G = Cumulative Illness Rating Scale - Geriatric version; WASO = Wake After Sleep Onset; L5 = Mean activity during least active five-hour period; M10 = Mean activity during most active ten hour period; WML = White matter lesions.
